# Environmental DNA Detects Endangered Texas Hornshell and Its Hosts

**DOI:** 10.1002/ece3.71397

**Published:** 2025-07-10

**Authors:** Daniel H. Mason, Heidi A. Henderson, Thomas W. Franklin, Michael K. Young, Jennifer E. Hernandez, Cory L. Engkjer, Joanna Hatt, Michael K. Schwartz

**Affiliations:** ^1^ National Genomics Center for Wildlife and Fish Conservation USFS Rocky Mountain Research Station Missoula Montana USA; ^2^ New Mexico Department of Game and Fish Santa Fe New Mexico USA

**Keywords:** blue sucker, freshwater mussel, gray redhorse, quantitative PCR, Rio Grande

## Abstract

Environmental DNA techniques are continuously demonstrating their efficiencies over traditional survey methods for detecting rare species. Unionids have a need for focused conservation efforts as they represent some of the most imperiled aquatic species in North America. Freshwater mussels are notoriously difficult to detect using traditional sampling techniques due to their cryptic morphological features, benthic habitat use, and low densities. Further, the persistence of freshwater mussel populations requires that viable fish host species be present during spawning. Therefore, improved conservation tools are needed to detect and monitor both the imperiled unionids as well as their associated fish host species. Here, we developed novel quantitative PCR (qPCR) assays for the detection of the endangered Texas hornshell ( *Popenaias popeii* ) and two of its' host species, the gray redhorse ( *Moxostoma congestum* ) and blue sucker ( *Cycleptus elongatus* ), which are also species of greatest conservation need in New Mexico. All qPCR assays were highly sensitive, species‐specific, and ultimately validated using eDNA samples collected from streams. These assays offer new tools for managers to detect, monitor, and conserve these imperiled species using environmental DNA.

## Abstract 2

We developed novel quantitative PCR (qPCR) assays for the detection of the endangered Texas hornshell ( *Popenaias popeii* ) and two of its' host species, the gray redhorse ( *Moxostoma congestum* ) and blue sucker ( *Cycleptus elongatus* ), which are also species of greatest conservation need in New Mexico. These assays offer new tools for managers to detect, monitor, and conserve these imperiled species using environmental DNA.

## 1 Introduction

Freshwater ecosystems account for less than 1% of the Earth's surface while supporting a disproportionately high number of species compared to marine or terrestrial habitats (Strayer and Dudgeon 2010). These biodiversity hotspots also overlap with highly concentrated areas of anthropogenic disturbance as freshwater is also critical to human well‐being. As such, freshwater ecosystems are among the most affected in the world, and declines in biodiversity are far greater than in terrestrial ecosystems (Ricciardi and Rasmussen 1999; Collen et al. 2014; WWF 2024). This is particularly true for freshwater mussels (Superfamily: Unionoidea) in North America as they represent one of the most imperiled and declining taxa across the planet due to habitat degradation, non‐native species, and impacted water quality (Haag 2012; Dobler et al. 2018; Aldridge et al. 2023). Further, freshwater mussels exhibit a complex life history in which immature individuals (glochidia) are obligate parasites of fish and often rely on an intact native fish community to develop into juvenile mussels. Without the appropriate fish host species present during spawning, the glochidia are not able to successfully disperse, and the longevity of the mussel population may be affected. Therefore, freshwater mussel declines may be tightly linked to changes in native fish communities. Direct sampling methods for both groups are time‐ and labor‐intensive, which is compounded when species are rare or occur at low densities. Additionally, the cryptic nature of freshwater bivalves can make them difficult to detect using conventional survey methods unless substantial effort is applied (Metcalfe‐Smith et al. 2000; Smith 2006). Consequently, improved methods are needed to sensitively and reliably detect both freshwater mussels and their host fish species.

The federally endangered Texas hornshell mussel ( *Popenais popeii* (Lea 1857); Federal Register 83 FR 5720) is a unionid bivalve native to the Rio Grande basin in North America with only six known populations (USFWS 2023). Similar to other freshwater mussel species, it is threatened by habitat modification such as decreased water quality due to livestock and agricultural practices and increased sedimentation as well as dewatering of habitat from groundwater extraction. Climate is also expected to affect Texas hornshell as stream temperatures and drought are likely to increase and precipitation is likely to decrease across their range (USFWS 2023). Reintroduction efforts occurred in the Delaware River in 2013 and 2015 to support the species' recovery but drying of several reaches necessitated moving mussels to the Black River (NMDGF et al. 2019). Texas hornshell mussels may be host generalists, as a laboratory study demonstrated a large suite of fish species could be infected with glochidia (Lang 2001, 2004), but field observations in their native range describe a more restricted set of species which carry glochidia under environmental conditions, as well as variation in the potential ecological significance of these hosts (Levine et al. 2012). Two large‐bodied fish species may be especially likely to demonstrate spatial variation in ecological host significance: the gray redhorse ( *Moxostoma congestum* (Baird & Girard, 1854)) and blue sucker ( *Cycleptus elongatus* (Lesueur, 1817)). Both fish are species of greatest conservation need in New Mexico (NMDGF 2016) and have similar ecological attributes (Levine et al. 2012).

Environmental DNA (eDNA) sampling is a technique that potentially can resolve much of the existing uncertainty about where and when Texas hornshell, blue sucker, and gray redhorse occur. Numerous studies have demonstrated the reliability and sensitivity of eDNA sampling for low abundance species (McKelvey et al. 2016; Franklin, Wilcox, et al. 2019; Robinson et al. 2019), including freshwater mussels (Dysthe et al. 2018; Rodgers et al. 2020; Preece et al. 2020). Additionally, eDNA sampling has the ability to simultaneously detect ecologically and genetically disparate taxa, including mussels and their hosts (Dokai et al. 2023), with the same efficiency as single species eDNA sampling. To address the lack of information on their distributions, coupled with the need to conserve these three species, we developed and validated novel quantitative PCR (qPCR) assays for the detection of the endangered Texas hornshell, the gray redhorse, and the blue sucker. Improving the efficacy of sampling for the three ecologically linked species will allow managers to design conservation programs which maintain viable populations of each species, ensure that there is sufficient temporal and spatial overlap during the breeding season between populations of Texas hornshell and populations of its host fishes, and allow for more intensive research into the ecology of these animals.

## 2 Materials and Methods

### 2.1 Assay Design and In Silico Evaluation

We developed separate eDNA assays for the detection of Texas hornshell (TXHS), blue sucker (CYEL), and gray redhorse (GYRH; Table 1). We selected loci for assay design by considering representation of the target species, representation of the sympatric confamilials, and whether the locus contained sufficient interspecific variation to support a species‐specific eDNA assay. The assays were developed in the NADH dehydrogenase (ND1), cytochrome b (Cytb), and cytochrome c oxidase subunit 1 (COI) mitochondrial gene regions, respectively. During *in silico* development, we downloaded gene sequences of target and sympatric non‐target species from GenBank (TXHS, *n* = 31; CYEL, *n* = 185; GYRH, *n* = 49; Tables 2, 3, 4). To better inform TXHS assay design, we produced additional ND1 sequences from Texas hornshell (*n* = 4) and Texas lilliput (*n* = 3) tissue DNA samples to supplement the data available from GenBank. We amplified a segment of the ND1 gene from these samples in 30 μL reactions using the Leu‐uurF and LoGlyR primers developed by Serb et al. (2003). To better inform GYRH assay design, we produced additional COI sequences from nine gray redhorse from the Delaware (*n* = 4) and Black (*n* = 5) rivers using the FishF1 and FishR1 primers (Bhattacharjee et al. 2012). Each reaction contained 1.4 μM of both the forward and reverse primer, 15 μL of 2X GoTaq G2 Green Master Mix (Promega), 2 μL (~26 ng) of DNA template, and 5.16 μL deionized water. To create a no‐template control, a well on the plate was filled with the same reagent mix, but with an additional 2 μL of deionized water instead of DNA template. We applied a touchdown PCR profile that involved: 94°C for 5 min, then 13 cycles of 94°C for 45 s, annealing at 57°C for 2 min, dropping 1°C each cycle (13th cycle annealing at 44°C), and extension at 72°C for 1 min, followed by 25 cycles of 94°C for 45 s, 55°C for 1 min, 72°C for 1 min, then proceeding to final extension at 72°C for 10 min, and a final hold at 12°C. The products were cleaned with ExoSAP‐IT PCR Product Cleanup Reagent kit (Life Technologies) and were shipped to Eurofins Genomics (Louisville, Kentucky, USA) to be bi‐directionally sequenced on an ABI 3730X sequencer. We trimmed, cleaned, and created consensus sequences from the forward and reverse reads from each sample using Sequencher v5.4.6 (Gene Codes Corporation). New mussel ND1 and gray redhorse COI sequences are available on GenBank (accessions PQ865907‐13; PQ872923‐31).

**TABLE 1:** Optimized reaction concentrations of assay components for use in qPCR analysis of environmental DNA (eDNA) samples collected for Texas hornshell (TXHS), blue sucker (*CYEL*), and gray redhorse (*GYRH*) assay validation.

Species	Assay component	Sequence (5′‐3′)	Tm (°C)	amplicon Length (nt)	Optimal concentration (nM)
TXHS	Forward A	GCACTTAGACTATGACAATTATTTCCATCT	58.7	105	450
	Forward B	CACTCAGACTATGACAATTATTTCCATCT	58.3		450
	Reverse	GGTTGTGTAGACAGTTAGAGAGGAGATACATA	59.8		900
	Probe	FAM‐CTTTCTTCTCAAATGGTTTTAGGA‐MGBNFQ	70		250
CYEL	Forward primer	GGCAACAGTCATCACAAACCTCT	59.1	177	900
	Reverse primer	GTGTAGGAAGAGCAGGTGAATGATAG	58.5		600
	Probe	FAM‐AGATAACGCAACACTGACACG‐MGBNFQ	70		250
GYRH	Forward primer	CGCCCATGCYTTCGTC	61	109	900
	Reverse primer	GGGGAATGCCATGTCTGG	58.2		900
	Probe	FAM‐ATRCCCATTTTAATTGG‐MGBNFQ	68		250

**TABLE 2:** Sequence data retrieved from the NCBI GenBank database for assay design and *in silico* mismatch assessment of the Texas hornshell assay (*n* = 38).

Species	Accessions	AP	Assay mismatches			
			Forward A	Forward B	Reverse	Probe
*Popenaias popeii*	NC050058; MK045091‐93; PQ865907‐10	0.965	1	1	0	0
*Cyrtonaias tampicoensis*	MK045056‐57	0.376	4	3	7	8
*Lampsilis teres*	MG030349; MK226711‐12	0.125	4	5	4	7
*Megalonaias nervosa*	AY158794; MH633571; MH633597	0.18	5	4	4	6
*Potamilus metnecktayi*	MK045105‐07	0.181				
*Potamilus purpuratus*	MT669685‐87	0.14	4	3	3	6
*Pyganodon grandis*	MG199751‐52	0.181	4	3	5	5
*Quadrula apiculata* (*quadrula*)	AY158805; MG030351; MH633590	0.456	6	6	6	12
*Truncilla cognata*	MK045120‐21	0.275	2	1	9	8
*Toxolasma parvum*	GU085381; MG030360	0.293	5	5	9	8
*Toxolasma texasiense*	MG020455; PQ865911‐13	0.272	3	4	3	5
*Uniomerus tetralasmus*	MG030355; MH633583; MH633605	0.254	5	4	4	5

*Note:* “N” refers to sample size, “AP” refers to eDNAssay assignment probability.

We aligned all sequences in MEGA 7 (Kumar et al. 2016) and visually identified candidate primer sites with the greatest number of nucleotide mismatches between the target and non‐target species. The alignments for each assay included data from all the sympatric confamilials to allow for the design of assays that will produce robust eDNA inferences within the Rio Grande basin. The TXHS assay involved using two forward primers to account for a single nucleotide polymorphism (SNP) without negatively affecting primer melt temperatures. The GYRH assay also needed to accommodate several SNPs in the target species, but in this case, we chose to incorporate degenerate nucleotides rather than design additional oligos. All three assays use a probe with a FAM‐labeled, minor‐groove‐binding, non‐fluorescent quencher (MGB‐NFQ) (Table 1). We counted mismatches between each assay component and each target and non‐target species' sequences to ensure the assay would be specific to its' respective target species. We utilized Primer Express 3.0.1 (Life Technologies) to adjust the length of each component for an optimized melt temperature. We then aligned the optimized candidate assay with the target and non‐target sequences and re‐analyzed the number of mismatches (Tables 2, 3, 4). We proceeded to analyze secondary oligonucleotide structures with the OligoAnalyzer web application (IDT). We used NCBI nucleotide BLAST (Ye et al. 2006) to further examine cross‐amplification potential with other species that inhabit the same environment as the target species. Any taxon that had > 95% shared sequence identity at both primer binding sites according to BLAST and was sympatric to the target species had its sequence data added to the alignment to examine cross‐amplification potential. Finally, we used eDNAssay (Kronenberger et al. 2022), a machine learning tool for predicting qPCR amplification from non‐target DNA template, to identify closely related confamilials that could produce false‐positive results from eDNA samples. We also applied the tool to identify confamilials from other river basins (not in the Rio Grande system) that would potentially be problematic if either the GYRH or CYEL assay was ever applied to a different study area. For this purpose, we applied an assignment probability threshold of 0.5 as indicating non‐target templates that were cause for concern, and then carried out further specificity validation in vitro for all three assays.

### 2.2 Experimental Validation

To optimize the primer concentrations for each assay, we ran each of the 16 possible pairwise combinations of four different primer concentration combinations (100, 300, 600, 900 nM), all with a probe concentration of 250 nM, in triplicate on a single plate, with each well containing ~0.4 ng of target genomic DNA, 7.5 μL Environmental Master Mix (Life Technologies), and a no assay control that replaced the assay with PCR grade water. We performed this test on the same instruments and with the same thermocycler conditions. We identified the optimal concentration of each assay by selecting the combination that produced the earliest mean threshold cycle (C_t_) and also demonstrated an exponential amplification phase.

Once we determined the optimized primer concentrations for each assay (900 nM: 900 nM TXHS and GYRH, 900 nM: 600 nM CYEL) we cleaned the qPCR products with a GeneJET PCR Purification Kit (ThermoFisher). To test assay efficiency in vitro, we quantified the cleaned products with a Qubit 2.0 fluorometer and made a standard curve with a five‐fold serial dilution. We then ran the standard curve with seven serial dilution levels in six replicates (31,250, 6250, 1250, 250, 50, 10, 2 copies/reaction) with a no‐template control run as an eighth set of six replicates. We identified the limit of detection (LOD) for each assay using the curve‐fitting method of Klymus et al. (2020), a probability of detection of 95%, and a standard curve with five dilution levels in eight replicates (250, 50, 10, 2, 0.4 copies/reaction) with a no‐template control run as a sixth level of eight replicates. Thermocycler conditions and instruments remained unchanged.

We tested assay specificity against Rio Grande taxa in vitro in single‐well reactions with a QuantStudio 3 Real‐time quantitative PCR (qPCR) instrument (Life Technologies) using tissue‐derived DNA extracts and gBlock (IDT) synthetic templates from selected nontarget species for which we were unable to obtain tissue. For the GYRH and CYEL assays, we also tested a representative portion of the allopatric confamilials which have assignment probabilities (APs) > 0.29 and < 0.8 to test for evidence that the eDNAssay threshold we selected (0.50) inadequately hedged against the risk of amplifications from non‐target templates. We stored tissue samples either in 95% ethanol or on chromatography paper prior to extraction. To reduce the likelihood of non‐target DNA contaminating the tissues, we rinsed them twice with a 10% sodium hypochlorite solution followed by two rinses with deionized water before continuing to DNA extraction with a DNeasy Tissue and Blood Kit (Qiagen Inc) using a modified protocol. Modifications to the protocol included an incubation of samples overnight at 56°C, an incubation at 70°C after the addition of Buffer AL, a final elution with 70°C warmed 1X TE followed by a 1 min room temperature incubation before centrifuging the elution column at max speed (21,100 × G). We quantified genomic DNA from extracted samples with a Qubit 2.0 fluorometer (Life Technologies) and diluted samples to a 0.1 ng μL^−1^ working concentration (Dysthe et al. 2018). We stored tissue DNA extracts and 0.1 ng μL^−1^ dilutions in a −30°C freezer until we used them for validation tests. We diluted gBlock fragments to 5000 copies reaction^−1^ for in vitro testing of templates which were not available as genomic DNA (Kronenberger et al. 2022). We set thermocycler conditions to 95°C for 10 min followed by 45 cycles of denaturation at 95°C for 15 s and an annealing step at 60°C for 1 min. qPCR occurred in 15‐μL reactions containing 7.5 μL Environmental Master Mix (Life Technologies), 450 nM of each Forward A and B primers for TXHS (900 nM for CYEL and GYRH), 900 nM Reverse primer for TXHS and GYRH (600 nM for CYEL), and 250 nM probe, 4 μL template DNA (~0.4 ng genomic DNA or 5000 gBlock copies) or PCR grade water for the NTC, and the remaining volume composed of PCR grade water.

All eDNA samples were collected by pumping up to 5 L of stream water through a 1.5 μm pore‐sized glass microfiber filter (GE HealthCare) using a peristaltic pump (GeoTech Environmental Equipment Inc.) following the collection protocol developed by Carim et al. (2016) and analyzed as in Dysthe et al. (2018). Occasionally in turbid water, up to three filters were required to filter up to 5 L. Used filters were individually placed in plastic bags with silica desiccant, packaged in envelopes labeled with relevant field information (i.e., date and sampling location), and mailed to the National Genomics Center for Fish and Wildlife Conservation (NGC; Missoula, MT).

Upon receipt of samples at the NGC, sampling data were cataloged, and samples were stored at −20°C until analysis. For each sample, eDNA was extracted from half of the sample filter using the Qiagen DNEasy Blood and Tissue Kit following a modified protocol described in Franklin, McKelvey, et al. (2019). The modified protocol doubles the volume of each of the following reagents: ATL, proteinase K, AL, and ethanol. After the second incubation, we separated the liquid from the filter by centrifugation in QIAShredder columns (Qiagen, Valencia CA, USA) and transferred it to DNeasy columns for the final two washes before eluting extracted DNA in 100 μL of warm TE (pH 8.0, 10 mM Tris, 0.1 mM EDTA; Integrated DNA Technologies). Each batch of extracted samples included an extraction from a clean, new filter as a negative control. The other half of the sample filter was retained and archived at −20°C. If more than one filter was used to collect the sample, lysate from all extracted filter halves for a given sample was spun through a single DNeasy column and washed from the column in a single elution. The filters were processed in four mixed batches of up to 23, with each batch including a negative extraction control as a test for systematically introduced contaminating DNA (Hutchins et al. 2021). Each batch was partially composed of filters from other NGC projects, and some samples had multiple filters, so extracting filters with this procedure produced a total of four negative extraction controls, but each control related to either two, five, or eight of the samples for this project. We used the optimized qPCR conditions from in vitro validation to analyze the samples in triplicate with the appropriate assay. We ran all eDNA samples with TaqMan Exogenous Internal Positive Control (IPC; Life Technologies) to detect any potential environmental contaminants that were inhibiting qPCR detection. The 96‐well plates also included triplicate runs of a positive tissue‐derived DNA sample (0.1 ng μL^−1^) and a triplicate no‐template control (NTC). An eDNA sample was considered inhibited if its mean IPC cycle (*C*_t_) was > 1 C_t_ higher than the mean IPC *C*_t_ from the plate NTC. We omitted standard curves from eDNA experiments because absolute quantification of eDNA concentration is uninformative for the purpose of assay validation. The 15‐μL reaction wells contained 7.5 μL Environmental Master Mix (Life Technologies), 450 nM of each forward A and B primers for TXHS or 900 nM forward primer for CYEL and GYRH, 900 nM reverse primer for TXHS and GYRH or 600 nM for CYEL, 250 nM probe (Table 1), 1.5 μL‐10X IPC assay, 0.3 μL‐50X IPC DNA, 4 μL template eDNA, or PCR grade water for the NTC, and the remaining volume with PCR grade water. A species was considered detected in an eDNA sample if its assay produced an exponential amplification curve with a *C*_t_ < 45 in more than one technical replicate. For samples with single‐well, exponential amplification curves with a *C*_t_ < 45, the species was considered detected if there were no immediately neighboring plate wells which also produced an amplification with the same assay. Samples with single‐well detections that neighbored other wells with detections were re‐analyzed, and the sample was only considered to have produced a detection if an amplification also occurred during re‐analysis.

**FIGURE 1:**
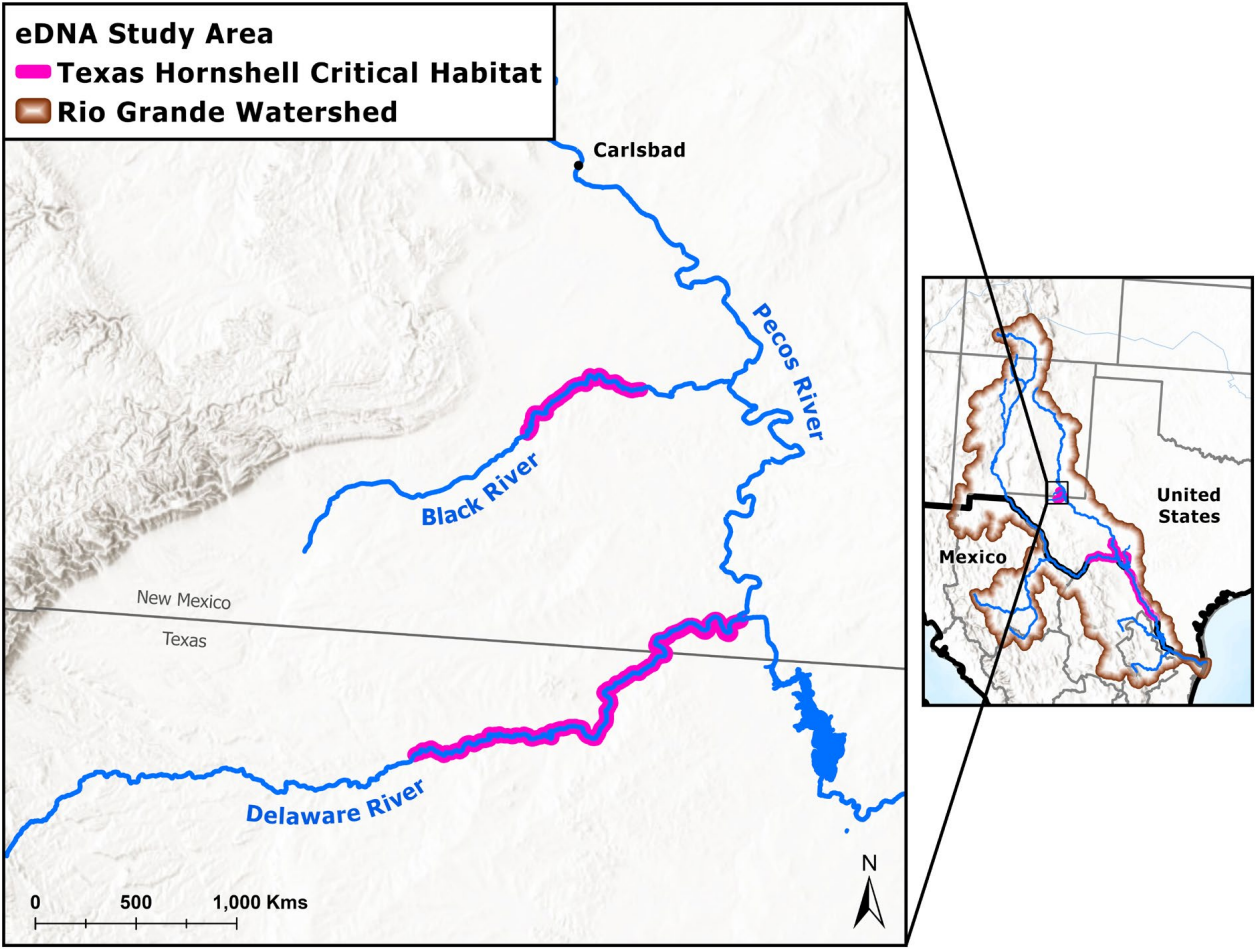
Map of the study area where expected positive and experimental eDNA samples were collected (Table 6), with an inset of the Rio Grande watershed (HUC13). Texas hornshell critical habitat (USFWS 2021) is shown in magenta.

We finished validation in situ by analyzing known positive eDNA samples for each target species with its respective optimized assay (Table 1), and additional samples from sites with unknown occupancy. Sampling materials, field equipment, and protocols were sent to the New Mexico Department of Game and Fish (NMDGF) for the collection of the eight unknown eDNA samples from streams in southeastern New Mexico. The NMDGF sampled three streams in the Rio Grande watershed between 19 October 2022 and 20 October 2022 (Table 6; Figure 1). The NMDGF also collected seven positive control eDNA samples from locations known to be occupied by Texas hornshell, gray redhorse, or blue sucker to validate the three eDNA assays: five on 29 March 2022 from the Black River near Carlsbad, NM, and two on 27 May 2022 from the Albuquerque Biological Park (ABQ BioPark); (Table 6). Two of the known positive samples from the Black River were also considered unknown samples for CYEL, as there was no prior information regarding the species' presence there (BLAR_01 and 02; Table 6).

## 3 Results

### 3.1 In Silico Validation

We observed a single nucleotide polymorphism (SNP) in the forward primer region of Texas hornshell sequences retrieved from GenBank. We developed two forward primers to accommodate the SNP, which resulted in both forward A and B having 1 nucleotide mismatch each with the target sequences but will allow the assay to amplify both haplotypes with equal efficiency and will maintain acceptable primer melt temperatures (Table 1). We observed similar SNPs in the GYRH forward and probe sites, but in this case were able to accommodate these with degenerate nucleotides rather than separately designed oligonucleotides (Table 1). Sequence data from Texas hornshell congeners are extremely limited, and the species has a highly restricted distribution, so we evaluated the TXHS assay with eDNAssay using only data from the other mussel taxa known from the Rio Grande (Supplemental Information). BLAST analysis of the CYEL assay revealed two *Gila* species with no nucleotide mismatch in the probe region of the candidate assay (Table 3). However, there were several mismatches with both forward and reverse primers. We used data from across Catostomidae to evaluate CYEL using eDNAssay and did not observe any confamilials with an assignment probability > 0.32, with one exception (Supplemental Information). The assay is a perfect match to the southeastern blue sucker ( *Cycleptus meridionalis* ), a species of conservation concern, native to Gulf of Mexico basins in the USA. Since *C. elongatus* and *C. meridionalis* do not co‐occur, and since the assay is very likely to exclude any other catostomid, the CYEL assay could also be useful for species‐specific detection of *C. meridionalis* from eDNA samples. Assessment of the GYRH assay revealed sufficient nucleotide mismatches between the assay and the non‐target species from the Rio Grande (Table 4), but eDNAssay produced assignment probabilities > 0.5 for twelve non‐target species of *Moxostoma* (Supplemental Information). We were unable to find evidence that any of these twelve species have a distribution which overlaps that of *M. congestum* (Page and Burr 2011). However, some of their ranges appear uncertain, especially the Mexican taxa. Caution is warranted if the GYRH assay is applied to eDNA sampling outside of the Rio Grande basin in the future.

**TABLE 3:** Sequence data from NCBI GenBank database used for assay design and *in silico* mismatch assessment of the blue sucker assay (*n* = 185).

Species	Accession	AP	Mismatches		
			Forward primer	Reverse primer	Probe
*Cycleptus elongatus*	AB126082; AF454868; JF799439; EF062361‐6, 9; EF062370, 2	0.868	0	0	0
*Carpiodes carpio*	AF454837; JN053177, 85, 87–8, 90, 93–94; JN053201, 8, 21–3, 37–39	0.065	5	4	3
*Carpiodes cyprinus*	JN053178‐9; JN053191, 5–7, 9 JN053203, 5, 9–18	0.065	5	3	2
*Carpiodes velifer*	JN053180‐2, 4, 6, 9; JN053192; JN053204, 62; JF799434; JX488762	0.065	5	4	3
*Ictiobus bubalus*	FJ226281, 5, 7–9; FJ226290, 9; FJ226300, 2–3, 6–9; FJ226340‐2	0.053	5	4	3
*Ictiobus cyprinellus*	FJ226256‐9; FJ226260‐9; FJ226270; FJ226286; FJ226291‐2	0.053	5	5	3
*Ictiobus niger*	FJ226271‐9; FJ226280, 2–4; FJ226309, 13–15; FJ226321, 5–6	0.053	5	4	3
*Hypentelium nigricans*	AF454909; AY253341‐2	0.314	6	2	1
*Thoburnia atripinnis*	AF454911; JF799530	0.15	5	7	2
*Erimyzon oblongus*	AF454876	0.06	7	6	4
*Erimyzon sucetta*	AF454878; KU697910	0.055	10	6	3
*Minytrema melanops*	AF454879; JF799447‐8	0.058	7	4	2
*Moxostoma albidum*	JF799491‐3	0.11	7	7	1
*Moxostoma anisurum*	AF454880‐1; JF799450	0.12	7	4	3
*Moxostoma breviceps*	AF180821; AF454888; JF799453	0.118	7	5	3
*Moxostoma carinatum*	AF454884; JF799455‐6	0.119	7	8	2
*Moxostoma congestum*	AF180820; AF522290‐1	0.111	8	8	1
*Moxostoma duquesnei*	AF454894‐5; JF799462	0.12	7	5	2
*Moxostoma erythrurum*	AF454886‐7; AY253421	0.14	7	6	2
*Moxostoma macrolepidotum*	AF454890; JF799473‐4	0.116	7	4	3
*Moxostoma mascotae*	AF454899; JF799520‐1	0.077	7	7	2
*Moxostoma pisolabrum*	AF454889	0.116	7	5	3
*Moxostoma poecilurum*	AF454896; AY253422; JF799479	0.133	8	4	2
*Moxostoma valenciennesi*	JF799487; AF454893	0.119	7	6	2
*Catostomus commersonii*	JF799435‐7	0.131	8	3	1
*Catostomus platyrhynchus*	JX488801‐2; KJ441248	0.153	6	6	3
*Catostomus plebeius*	JX488803‐4	0.165	7	6	4
*Gila nigra*	KF514210; JX443027‐8	n/a	7	7	0
*Gila robusta*	FJ913816; JX443033‐5; KF514254‐5	n/a	5	7	2
*Gila intermedia*	JX443036; KF514194	n/a	7	7	0
*Campostoma oligolepis*	DQ486794; DQ486802, 12, 15	0.097	8	6	3
*Campostoma anomalum*	DQ486795‐9	n/a	5	4	1

*Note:* “*n*” refers to samples size, “AP” refers to eDNAssay assignment probability, “n/a” indicates the species' sequence was not assessed with eDNAssay because it is not a confamilial.

**TABLE 4:** Sequence data from NCBI GenBank database used for oligonucleotide design and *in silico* mismatch assessment of the gray redhorse assay (*n* = 58).

Species	Accession	AP	Mismatches		
			Forward primer	Reverse primer	Probe
*Moxostoma congestum*	HQ573198; HQ573331; JN027278‐9; JN027280‐2; HQ573187; PQ872923‐31	0.853	0	0	0
*Catostomus commersonii*	KX145116; KX145335; KX145540; KX145592; EU523931; EU524476‐9; EU524480‐4; JN024912	0.066	2	4	5
*Carpiodes carpio*	JN024862‐6	0.217	3	3	3
*Cycleptus elongatus*	JN025162‐3; KF929801	0.237	3	3	2
*Ictiobus bubalus*	JN026913‐6; KF929996	0.166	3	2	3
*Moxostoma macrolepidotum*	KX145069; KX145338; KX145484; KX145493; EU524889; EU524890‐2; EU524896‐8; EU524901‐2	0.388	2	2	0

*Note:* “n” refers to sample size, “AP” refers to eDNAssay assignment probability.

### 3.2 Experimental Validation & eDNA Results

Assay testing did not amplify any DNA templates from sympatric non‐targets, and amplified all the target samples tested (Table 5). The TXHS and CYEL assays appear to be highly specific. There are three allopatric confamilials which are nearly certain to amplify with GYRH, based on their eDNAssay APs (*Moxostoma albidum, M. duquesnii, M. poecilurum* (Supporting Information—S1)). We confirmed in vitro that there are four other confamilials which may be amplified by GYRH (Table 5). This is not ideal for an eDNA assay, but we could not design a globally specific assay for gray redhorse with the available data, and GYRH does appear to be specific against sympatric non‐targets. Optimal primer concentrations are reported below (Table 1). The TXHS assay amplified a standard curve with the following characteristics: Slope = −3.419, y‐intercept = 41.892, *R* ^2^ = 0.993, Efficiency % = 96.087, and Limit of Detection (LOD) = 3.4 copies/reaction. The CYEL assay produced a curve with slope = −3.475, y‐intercept = 40.009, *R* ^2^ = 0.993, efficiency % = 93.997, and LOD = 21.9 copies/reaction, with an amplification of 5/6 wells at 2 copies/reaction. We also observed efficient and sensitive performance from the GYRH standard curve: Slope = −3.415, y‐intercept = 38.176, *R* ^2^ = 0.989, efficiency % = 96.271, LOD = 1.2 copies/reaction. Analysis of the known positive eDNA samples resulted in amplification of all known positives for each assay (Table 6). There was no amplification of extraction negative controls or qPCR NTCs, and the presence of PCR inhibitors was not detected in any of the eDNA samples.

**TABLE 5:** Species tested and results for tissue‐derived DNA samples and gBlocks used during in vitro testing for taxonomic specificity of the Texas hornshell (*n* = 27), blue sucker (*n* = 66), and gray redhorse (*n* = 56).

Family	Species	Common name	Origin	*n*; TXHS	*n*; CYEL	*n*; GYRH
Unionidae	*Popenaias popeii*	Texas hornshell	NM	7; +	NT	NT
	*Potamilus purpuratus*	Bleufer	TX	2; −	NT	NT
	*Pyganodon grandis*	Giant floater	TX	1; −	NT	NT
	*Toxolasma parvum*	Lilliput	TX	2; −	NT	NT
	*Quadrula quadrula*	Mapleleaf	TX	2; −	NT	NT
	*Truncilla cognata*	Mexican fawnsfoot	TX	2; −	NT	NT
	*Potamilus metnecktayi*	Salina mucket	TX	2; −	NT	NT
	*Cyrtonaias tampicoensis*	Tampico pearlymussel	TX	2; −	NT	NT
	*Toxolasma texasiense*	Texas lilliput	TX	2; −	NT	NT
	*Megalonaias nervosa*	Washboard	TX	3; −	NT	NT
	*Lampsilis teres*	Yellow sandshell	TX	2; −	NT	NT
Catostomidae	*Cycleptus elongatus*	Blue sucker	TX	NT	10; +	2; −
			NM	NT	1; +	1; −
	*Moxostoma congestum*	Gray redhorse	NM	NT	9; −	9; +
	*Catostomus platyrhynchus* †	Mountain sucker	WY	NT	1; −	2; −
	*Catostomus plebeius*	Rio Grande sucker	NM	NT	13; −	13; −
			TX	NT	1; −	1; −
			CO	NT	NT	3; −
	*Carpiodes carpio*	River carpsucker	MT	NT	1; −	1; −
	*Ictiobus bubalus*	Smallmouth buffalo	MT	NT	4; −	2; −
	*Catostomus commersonii*	White sucker	NM	NT	8; −	2; −
	Hypentelium nigricans*†	Northern hogsucker	AF454909.1	NT	1; −	NT
	Moxostoma austrinum*†	Mexican redhorse	JN027268.1	NT	NT	1; +
	Moxostoma valenciennesi*†	Greater redhorse	EU524150.1	NT	NT	1; −
	Moxostoma lachneri*†	Greater jumprock	JN027291.1	NT	NT	1; +
	Moxostoma breviceps*†	Smallmouth redhorse	JN027269.1	NT	NT	1; +
	Moxostoma anisurum*†	Silver redhorse	EU524146.1	NT	NT	1; −
	Moxostoma erythrurum*†	Golden redhorse	EU524867.1	NT	NT	1; +
	Thoburnia rhothoeca*†	Torrent sucker	JN028426.1	NT	NT	1; −
	Catostomus catostomus*†	Longnose sucker	OR814557.1	NT	NT	1; −
	Hypentelium roanokense*†	Roanoke hogsucker	JN026848.1	NT	NT	1; −
	Hypentelium etowanum*†	Alabama hogsucker	JN026826.1	NT	NT	1; −
	Moxostoma macrolepidotum*†	Shorthead redhorse	EU524149.1	NT	NT	1; −
	Moxostoma rupiscartes*†	Striped jumprock	JN027314.1	NT	NT	1; −
	Moxostoma robustum*†	Robust redhorse	JN027311.1	NT	NT	1; −
	Moxostoma ariommum*†	Bigeye jumprock	JN027266.1	NT	NT	1; −
	Erimyzon tenuis*†	Sharpfin chubsucker	JN025449.1	NT	NT	1; −
	Erimyzon oblongus*†	Creek chubsucker	AP011228.1	NT	NT	1; −
	Catostomus nebuliferus*†	Nazas sucker	EU668538.1	NT	NT	1; −
Cyprinidae	*Cyprinus carpio*	Common carp	WA	NT	3; −	NT
Leuciscidae	*Gila intermedia* †	Gila chub	NM	NT	2; −	NT
	*Gila robusta* †	Roundtail chub	NM	NT	1; −	NT
			AZ	NT	10; −	NT
			CO	NT	1; −	NT

*Note:* Species whose DNA was represented by a gBlock are marked with a “*”. Origin refers to the state of collection within the United States for tissue DNA. Origins of gBlocks are designated with the accessions of the NCBI GenBank sequences which were used as inputs for gBlock synthesis. “+” indicates that we visualized amplification with the assay; “−” indicates we did not visualize amplification, and “NT” indicates that DNA from the species was not tested in vitro with the assay. To more completely describe the performance of the assays, we tested DNA from several taxa which are not known to occur in sympatry with any of the three target species, but which are confamilials. These taxa are marked with a “†”.

**TABLE 6:** Collection information of known positive eDNA samples used for in situ testing of the Texas hornshell (*n* = 5), blue sucker (*n* = 2), and gray redhorse (*n* = 7) assays, and also samples which were unknowns (*n* = 8).

Date collected	Feature name	Sample	TXHS detected	CYEL detected	GYRH detected
3/29/2022	Black River	NM_032922_BLAR_01	Y*	Y	Y*
3/29/2022	Black River	NM_032922_BLAR_02	Y*	N	Y*
3/29/2022	Black River	NM_032922_BLAR_03	Y*	—	Y*
3/29/2022	Black River	NM_032922_BLAR_04	Y*	—	Y*
3/29/2022	Black River	NM_032922_BLAR_05	Y*	—	Y*
5/27/2022	ABQ BioPark	NM_052722_TANK_01	—	Y*	Y*
5/27/2022	ABQ BioPark	NM_052722_TANK_02	—	Y*	Y*
10/19/2022	Delaware River	NM_101922_DELR_01	N	N	Y
10/19/2022	Delaware River	NM_101922_DELR_02	N	N	N
10/19/2022	Black River	NM_101922_BLAR_03	N	N	N
10/19/2022	Black River	NM_101922_BLAR_04	N	N	Y
10/19/2022	Black River	NM_101922_BLAR_05	Y	N	Y
10/20/2022	Pecos River	NM_102022_PECR_06	N	N	Y
10/20/2022	Pecos River	NM_102022_PECR_07	N	N	Y
10/20/2022	Pecos River	NM_102022_PECR_08	N	N	Y

*Note:* The species was considered detected if the corresponding assay amplified DNA in at least one of the qPCR replicates. “Y” indicates that we visualized amplification with the assay; “N” indicates we did not visualize amplification. Results produced from known positives are indicated with “*.” Samples with results of “‐” indicate that no analysis was performed with the assay.

We analyzed eight additional eDNA samples with the newly validated assays, collected in October 2022, from three streams in New Mexico, and re‐analyzed two for CYEL (Table 6). The samples were collected at locations which had uncertain occupancy status for the three species. We detected all three taxa in the Black River, and additionally detected gray redhorse in the Pecos and Delaware Rivers (Table 6).

## 4 Discussion

We have demonstrated that all three qPCR assays are able to detect their targets from environmental samples, amplify efficiently, and are sensitive to low quantities of DNA. The evidence from assay validation suggests that the Texas hornshell, blue sucker, and gray redhorse assays are well suited for eDNA sampling in the Pecos basins and could be applied more broadly in the Rio Grande basin. Application of the blue sucker assay outside of the Rio Grande basin likely warrants further in vitro testing to confirm its generality, as populations from other portions of the fish's extensive range may contain unsampled haplotypes that could affect assay performance. Further eDNA sampling for these three species should incorporate accepted best practices, and results from these assays should continue to be evaluated as scientific understanding of these species improves, especially as more genetic and occurrence data become available.

We have focused here on carefully describing the development of a trio of eDNA assays, and our study does not address a number of factors which are important for the application of eDNA assays to systematic survey efforts (Thalinger et al. 2021). The three assays are well validated, but our in situ testing effort was not exhaustive, and our sampling design does not allow us to empirically estimate probabilities of detection or identify specific factors in our study area which might influence this. More expansive eDNA sampling within the study watersheds and a concerted effort to account for any variation in detection probability could allow investigators to use eDNA data to make more useful biological inferences across space and time (Goldberg et al. 2016). There remain important biological features (e.g., age structure, sex ratio, behavioral variation, species interactions) which aquatic eDNA sampling cannot currently measure, and when these features are of interest, eDNA sampling should be applied as a complementary approach to direct observation methods, not as a replacement.

Though the data from traditional surveys can be invaluable for some management questions, successfully managing freshwater mussels necessitates data from both the mussels as well as the fish community (Modesto et al. 2017). This can effectively double the time, labor, and costs needed if independent surveys for each taxonomic group are required. Environmental DNA sampling represents an advantageous methodology in which the field collection (i.e., filtering water) can be less biased among taxa, demonstrating that freshwater mussel DNA can be reliably detected from water samples originally collected for delineating other aquatic species (Dysthe et al. 2018; Rodgers et al. 2020). Further, the assays developed here could be applied to previously collected eDNA samples to retroactively detect the species without re‐sampling (Franklin, Wilcox, et al. 2019). Efforts to reintroduce mussel populations could potentially benefit from using these tools to identify reaches which support host fish prior to re‐stocking with hornshell. Population augmentation or monitoring efforts may also benefit from eDNA methods if sampling can be used to more sensitively detect historic populations or natural recolonizations while the density of individuals is very low. As native freshwater mussel populations continue to decline across much of North America, this study enables the application of an efficient, non‐invasive sampling approach which can simultaneously detect fish hosts and the bivalves of conservation concern which rely on them.

## Author Contributions

**Daniel H. Mason:** conceptualization (equal), data curation (lead), formal analysis (lead), funding acquisition (supporting), methodology (equal), writing – original draft (lead), writing – review and editing (equal). **Heidi A. Henderson:** data curation (equal), formal analysis (equal), methodology (equal), writing – original draft (lead), writing – review and editing (equal). **Thomas W. Franklin:** conceptualization (equal), funding acquisition (lead), methodology (equal), project administration (lead), writing – original draft (supporting), writing – review and editing (equal). **Michael K. Young:** conceptualization (equal), funding acquisition (equal), investigation (lead), project administration (equal), resources (lead), writing – original draft (supporting), writing – review and editing (equal). **Jennifer E. Hernandez:** funding acquisition (supporting), project administration (lead), resources (supporting), writing – review and editing (equal). **Cory L. Engkjer:** formal analysis (equal), methodology (equal), writing – review and editing (equal). **Joanna Hatt:** conceptualization (equal), investigation (equal), resources (equal), writing – review and editing (equal). **Michael K. Schwartz:** conceptualization (equal), funding acquisition (equal), investigation (equal), resources (equal), supervision (lead), writing – review and editing (equal).

## Conflicts of Interest

The authors declare no conflicts of interest.

## Supporting information


**Appendix S1.** Supporting Information.

## Data Availability

The results from eDNAssay analyses for each assay are available as Supporting Information—S1. The mussel and redhorse sequence data produced by this study are available on NCBI GenBank.
